# A frontotemporal dementia–like phenotype in schizophrenia: links to striatal dopamine and iron accumulation

**DOI:** 10.1038/s41386-026-02426-x

**Published:** 2026-04-25

**Authors:** Xuanhan Chang, Linda Bryant, Sergio Mena Ortega, Oliver Howes, Jan Sedlacik, Mattia Veronese, Matthias L. Schroeter, Paris Alexandros Lalousis, Nikolaos Koutsouleris, Luke Vano, Fiona Coutts

**Affiliations:** 1 Institute of Psychiatry, Psychology & Neuroscience, King’s College London, London, UK; 2 Mansfield Centre for Innovation - MR Facility, MRC Laboratory of Medical Sciences, Hammersmith Hospital, London, UK; 3 Max Planck Institute for Human Cognitive and Brain Sciences, Leipzig, Germany; 4 Clinic for Cognitive Neurology at University Leipzig, Leipzig, Germany; 5 Department of Psychiatry and Psychotherapy, Ludwig-Maximilian-University, Munich, Germany; 6 Max-Planck Institute of Psychiatry, Munich, Germany; 7 DZPG (German Centre for Mental Health), Partner site Munich/Augsburg, Munich, Germany; 8 Centre for Addictions and Mental Health, Toronto, ON Canada

**Keywords:** Schizophrenia, Dementia

## Abstract

Some schizophrenia patients share characteristics with behavioral variant frontotemporal dementia (bvFTD) including gray matter volume (GMV) similarities, suggesting overlapping brain mechanisms that may contribute to disease heterogeneity in schizophrenia. However, it is not yet understood whether schizophrenia patients with a bvFTD-like GMV signature also show additional features of bvFTD. Seventy-six patients with schizophrenia (mean age 31.2 years; 71% male) from a previous clinical trial (NCT04038957) underwent structural magnetic resonance imaging (MRI) to measure GMV. Healthy controls (*n* = 79) were used for age-related dynamic standardisation. Subsets of patients also completed [¹⁸F]-DOPA positron emission tomography (PET; *n* = 40) for striatal dopamine synthesis, neuromelanin-sensitive MRI (NM-MRI; *n* = 68) for midbrain dopaminergic integrity, and quantitative susceptibility mapping (QSM; *n* = 69) for brain iron accumulation. A previously validated machine-learning–based GMV classifier quantified bvFTD-like pattern expression in each schizophrenia patient. Associations with neurochemical, clinical, and medication measures were examined using correlation analyses. Higher bvFTD scores were associated with lower striatal dopamine synthesis capacity (*r* = –0.343, *p* = 0.032) and higher striatal QSM values (*r* = 0.282, *p* = 0.020), but showed no significant association with QSM or NM-MRI values in the dopaminergic midbrain (*p* = 0.903 and *p* = 0.102, respectively). No significant associations were found with negative symptom severity or with medication. Schizophrenia patients expressing a stronger bvFTD-like GMV pattern show lower striatal dopamine synthesis and elevated striatal iron, both hallmark features of bvFTD. This contrasts with the hyperdopaminergic model of schizophrenia and suggests distinct, potentially neurodegenerative mechanisms.

## Introduction

Schizophrenia is a heterogeneous disorder with diverse symptom profiles, illness courses, and biological features [1–3]. While often viewed as a neurodevelopmental condition, accumulating evidence suggests that neurodegenerative processes may also be present, including progressive gray matter volume (GMV) loss and cognitive decline [4–7]. This perspective reflects Kraepelin’s early notion of dementia praecox [8, 9], and aligns with evidence of potential overlaps between schizophrenia and frontotemporal dementia (FTD), particularly its behavioral variant (bvFTD) [10].

FTDs arise from neuronal protein accumulation and dysfunction of the protein-degradation pathways [11]. In bvFTD, early frontotemporal protein aggregation leads to cortical atrophy and behavioral changes similar to the negative and cognitive symptoms of schizophrenia, such as anhedonia and executive dysfunction [12, 13]. This means bvFTD is frequently misdiagnosed as schizophrenia [10, 14]. bvFTD and schizophrenia also share genetic risk, including Chromosome 9 open reading frame 72 (C9orf72) [15], and are associated with similar GMV reductions in the prefrontal cortex, anterior cingulate, insula, and temporal regions [16, 17] as well as neuroinflammation [18, 19]. Furthermore, individuals with schizophrenia are 2–5 times more likely to develop dementia compared with the general population [20]. Taken together these findings suggest that there may be shared mechanisms between these two disorders.

To determine whether a shared brain signature exists across bvFTD and schizophrenia, Koutsouleris et al. [21] applied a machine-learning model that was originally trained to distinguish bvFTD patients from healthy controls using GMV to schizophrenia patients. They found that a subset of individuals with schizophrenia expressed a similar frontoinsular–limbic alteration pattern, indicating a bvFTD-like structural pattern within schizophrenia that may differ from the traditional neurodevelopmental view. This GMV pattern was predictive of functional outcomes in clinical high risk and depression. On the other hand, no shared pattern was observed when applying an Alzheimer’s disease (AD) model to the schizophrenia cohort, nor was the AD brain phenotype predictive of functional outcomes in clinical high risk and depression, suggesting this overlap is specific to bvFTD. However, it remains unclear whether schizophrenia patients expressing this pattern also exhibit other biological features characteristic of bvFTD.

In bvFTD, reduced striatal dopaminergic function is observed within corticostriatal circuits [22, 23]. In contrast, schizophrenia is usually characterized by elevated striatal dopamine synthesis, although heterogeneity exists: individuals with treatment-resistant schizophrenia (TRS) or predominant negative symptoms often show normal or even reduced dopaminergic function [24–26]. A similar pattern is seen in neuromelanin-sensitive MRI (NM-MRI) studies indexing neuromelanin in the substantia nigra and ventral tegmental area (SN-VTA), a byproduct of dopamine synthesis [27]. Specifically, treatment-responsive schizophrenia is associated with higher SN-VTA NM-MRI contrast-to-noise ratios (NM-CNRs) [28], whereas TRS shows lower NM-CNRs than controls [29].

Brain iron dysregulation has also been implicated in bvFTD, with ferroptosis and iron-mediated oxidative damage being linked to GMV loss [11]. In vivo brain iron can be assessed using quantitative susceptibility mapping (QSM) MRI to estimate magnetic susceptibility (χ), which strongly correlates with subcortical iron levels [30]. Meta-analytic findings from case–control studies using QSM in psychosis suggest that basal ganglia iron levels are lower in first-episode psychosis, with striatal heterogeneity driven by elevated iron in chronic schizophrenia [28].

These findings raise the possibility that individuals showing a bvFTD-like pattern in schizophrenia may have a distinct neurochemical profile, with reduced striatal dopamine synthesis and brain iron accumulation. This potential relationship might explain some of the biological heterogeneity of schizophrenia and may guide mechanism-based approaches to diagnosis and treatment. Thus, the current study aimed to characterize the neurochemical correlates of the bvFTD-like structural pattern observed in schizophrenia. We used data from previously published analyses showing that patients with schizophrenia had lower χ in the striatum and SN-VTA but elevated SN-VTA NM-CNR than controls [31, 32]. In patients with schizophrenia, we quantified bvFTD-like pattern from structural MRI (sMRI), striatal dopamine synthesis capacity (K_i_^cer^) using [¹⁸F]-DOPA positron emission tomography (PET), SN-VTA NM-CNR, and basal ganglia χ using QSM. Our primary hypotheses were that greater bvFTD-like structural pattern expression would be associated with lower striatal K_i_^cer^, lower SN-VTA NM-CNR, and lower χ in the striatum and SN-VTA. Exploratory analyses examined the PET and QSM associations within the striatal subregions.

## Materials and methods

### Participants

The data used in this study were drawn from Vano et al. (NCT04038957) [31] and assessed through the Department of Psychosis Shared Database Initiative (DPSDI), where all MRI, PET, NM-MRI, and QSM data had been fully preprocessed prior to analysis. Ethical approval was granted by the Office for Research Ethics Committees in Northern Ireland and the Administration of Radioactive Substances Advisory Committee for a clinical trial [33]. The current article only presents baseline data. Patients with schizophrenia (aged 18–45 years) were recruited from community mental health teams in London, United Kingdom. Diagnoses were confirmed by a study psychiatrist using the Structured Clinical Interview for DSM-5 (SCID-5) [34]. Exclusion criteria included comorbid psychiatric disorders, neurological illness, and current or past substance or alcohol use disorder. This was not a first-episode cohort, the mean illness duration was 2.7 years. Patients were either unmedicated (21%) or taking a single antipsychotic (79%) at the time of scanning. Individuals with current or previous clozapine use were excluded due to its distinct effects on dopaminergic function [25].

Healthy controls (aged 18–45 years) were recruited from the same geographic area via local advertisement and had no history of psychiatric or neurological illness. Controls underwent structural MRI scanning but were not included in case–control statistical analyses. Instead, their data served as a normative reference for dynamic standardization during external model application.

### Measures

#### Clinical measures

All patients with schizophrenia underwent clinical assessment using the Positive and Negative Syndrome Scale (PANSS) [35]. Participant age, age of illness onset and current medication status were also recorded, with doses converted to chlorpromazine equivalents via the method outlined by Leucht et al. [36]. Clinical and demographic characteristics are summarized in Table 1.

**Table 1:** Demographics, experimental variables, and clinical measures.

Variables	Schizophrenia patients, *n* = 76
Demographics	
Sex, male	54 (71%)
Age, years	31.2 (7.0)
Self-reported ethnicity	
Asian	9 (12%)
Black	40 (53%)
Mixed ethnicity	3 (4%)
White	24 (32%)
Neuroimaging measures	
No. With PET	41
No. With PET passing QC	40
Days between MRI and PET	12.4 (13.9)
Injected activity, MBq	141.5 [71]
No. With NM-MRI	71
No. With NM-MRI passing QC	68
No. With QSM	76
No. With QSM passing QC	69
Clinical measures	
PANSS total	73.3 (13.5)
PANSS positive	17.4 (5.6)
PANSS negative	20.3 (5.6)
PANSS general	35.7 (6.8)
Age of onset, years	26.7 (7)
Duration of illness, years	2.7 [2.7]
Current medication	
Olanzapine	19 (25%)
Aripiprazole	21 (28%)
Risperidone	7 (9%)
Paliperidone	7 (9%)
Lurasidone	3 (4%)
Flupentixol	2 (3%)
Lithium carbonate	1 (1%)
Unmedicated	16 (21%)
Chlorpromazine daily equivalent doses, mg	300.4 (150.2)

Results are expressed as n (%), mean (SD), or median [interquartile range].

*PANSS* Positive and Negative Syndrome Scale, *PET* positron emission tomography, *NM-MRI* neuromelanin-sensitive MRI, *QSM* quantitative susceptibility mapping, *QC* quality control.

### Neuroimaging acquisition

This protocol has been described in previous publications [31, 32]. All patients and healthy controls underwent MRI scanning on a 3 T system (MAGNETOM Prisma, Siemens Healthineers, Erlangen, Germany) equipped with a 64-channel receive-only head coil. High-resolution T1-weighted structural images were acquired using a 3D magnetization-prepared rapid gradient-echo (MPRAGE) sequence (TR = 2300 ms; TE = 2.91 ms; flip angle = 9°; bandwidth = 140 Hz/pixel; acceleration factor = 2; 176 sagittal slices; isotropic voxel size = 1 mm³; acquisition time = 5 min 35 s). An axial 2D gradient echo sequence with a magnetization transfer pulse was used for the NM-MRI (TR = 359 ms; TE = 2.85 ms; flip angle = 40°; bandwidth = 320 Hz/pixel; 5 averages; 12 axial slices; voxel size = 0.8 × 0.8 × 2 mm³; acquisition time = 5 min 50 s). A 3D gradient recall echo sequence was used to generate the QSM maps (TR = 50 ms, first TE at 5.84 ms with 8 subsequent echoes 4.79 ms apart, flip angle = 15°, 144 sagittal slices with the voxel size = 1 × 1 × 1 mm^3^; acquisition time = 8 min 5 s).

Only schizophrenia patients underwent PET imaging. Those who declined completed the study after MRI. PET-MR scans were completed on a SIGNA GE 3 T PET/MR scanner and followed a standard protocol [37, 38]. Participants fasted for 6 h (water only) and abstained from smoking for 4 h before scanning. To enhance [¹⁸F]-DOPA signal-to-noise ratio, 150 mg carbidopa and 400 mg entacapone were administered orally one hour before radiotracer injection [39]. A zero-time echo scan was acquired for attenuation correction, followed by an MR localizer. Up to 150 MBq of [¹⁸F]-DOPA was diluted in saline and injected over 20 s, followed by a 10 mL saline flush. Emission data were collected over 95 min and reconstructed into 26 time frames: one 30-s background frame, four 60-s frames, three 120-s frames, three 180-s frames, and fifteen 300-s frames.

### Data analysis

#### MRI preprocessing

sMRI scans were processed using the Computational Anatomy Toolbox (CAT12, version 1207; http://dbm.neuro.uni-jena.de/cat12/), implemented in Statistical Parametric Mapping (SPM12) for MATLAB [40]. Preprocessing included bias correction, tissue segmentation, and DARTEL normalization to MNI space [41]. Image quality was assessed using CAT12’s weighted interquartile range, and scans below the 80th percentile were excluded [42].

Rigid coregistration of the NM-MRI image to the sMRI space was completed using Advanced Normalization Tools 2.4.0 (ANTs) [43]. Each participant’s first echo magnitude image from the MPRAGE data was non-linearly warped into their sMRI space using a coarse 15-mm spline grid with NiftyReg. A midbrain atlas containing crus cerebri and SN-VTA masks (available from: https://github.com/lukevano/KCL_Neuromelanin-MRI) was moved from the MNI into NM-MRI and QSM space by applying the relevant affine transformation-matrices and warp-fields generated from the previous steps. Labels for the caudate nucleus, putamen, and nucleus accumbens from the CerebrA brain atlas were moved in the QSM space by the same process.

Each participant’s NM-MRI image was converted to an NM-CNR map by using the following equation for each voxel:

$$\frac{{voxel}\,{intensity}-{mode}\,{crus}\,{cerebri}\,{voxel}\,{intensity}}{{mode}\,{crus}\,{cerebri}\,{voxel}\,{intensity}}$$

The mode crus cerebri intensity was calculated via a kernel-smoothing-function fit to a histogram of voxels within the crus cerebri mask. These NM-CNR maps were quality controlled (QC) by steps outlined by Salzman et al. [44].

To generate the QSM images, a whole-brain mask was initially created from the magnitude image of the earliest echo in the 3D MPRAGE acquisition using FSL’s Brain Extraction Tool (BET) [45]. Phase data from every echo were then processed with the Fit_ppm_complex.m function in the MEDI toolbox to estimate voxelwise frequency offsets [46]. These estimates were extracted from a slightly eroded version of the BET-derived mask (roughly 95% of its original size) and converted to local field shifts using the projection-onto-dipole-fields approach [47]. QSM images were subsequently reconstructed from these local fields with an iterative Tikhonov-regularised dipole inversion algorithm [48].

#### PET preprocessing

[¹⁸F]-DOPA PET images were processed using a standard pipeline [37, 38] consistent with Vano et al. [31]. An atlas of striatal subdivisions (limbic, associative, sensorimotor) and cerebellum [48] was transformed from MNI to native PET space. Dopamine synthesis capacity (K_i_^cer^) was determined using the cerebellum as reference via the Patlak–Gjedde method [49]. Analyses used an in-house MATLAB script incorporating Piwave [50], the FDOPA package, and SPM12. QC was completed following the procedures described in Nordio et al. [51]. Briefly, each dynamic frame was rigidly coregistration to the 15-min frame. Datasets showing substantial residual misalignment after correction (e.g., clear between-frame displacement or blurring/distortion) were excluded for motion artefact. Placement of the striatal and cerebellar atlas was checked on each participant’s PET image. If misaligned or if the striatal values were non-physiological (negative or out of physiological range), this data was also excluded.

#### Pre-trained bvFTD classifier

A published bvFTD classifier was re-trained in the current study using NeuroMiner (v1.2; https://neurominer-git.github.io/NeuroMiner_1.2/intro.html) [52], the same software version used for later model application, to ensure full pipeline compatibility. The model is a linear support vector machine originally developed by Koutsouleris et al. [21] to distinguish 108 patients with bvFTD from 40 age-matched healthy controls across four imaging sites.

The classifier was developed using GMV maps that were preprocessed with CAT12 as described above, implemented in SPM12, following the standard voxel-based morphometry pipeline. This included Spatially Adaptive Non-Local Means (SANLM) denoising, adaptive segmentation (AMAP), partial volume correction, and high-dimensional DARTEL registration to MNI space. The GMV maps were modulated by Jacobian determinants, resliced to 3-mm isotropic voxels, and adjusted for cohort and age effects. Model optimization and validation were performed using a repeated nested 10×10 cross-validation framework with principal component analysis–based dimensionality reduction and covariate control for sex, image quality, and total GM volume.

The spatial signature of the bvFTD classifier, including reliable volume reductions in prefrontal, orbitofrontal, insular, anterior cingulate, medial temporal, and striatal regions, was characterized in detail in the original publication by Koutsouleris et al. [21] using cross-validation ratio mapping.

#### External application

Before applying the model to our cohort of schizophrenia patients and healthy controls, a dynamic voxel-wise standardization pipeline was implemented. to minimize domain shift between the discovery cohorts and our sample [21]. Gray-matter maps were z-scored using voxel-wise distributions from our age-matched healthy controls, and unreliably estimated voxels were masked, enabling age- and site-independent application of the bvFTD classifier.

External application of the model was also conducted in NeuroMiner v1.2. The bvFTD classifier was applied to sMRI data from schizophrenia patients (*n* = 76) and healthy controls (*n* = 79) to derive a continuous decision score (“bvFTD score”) quantifying the similarity of each participant’s brain alteration pattern to that observed in bvFTD. Positive scores indicated more bvFTD-like alteration, whereas negative scores indicated control-like patterns.

#### Statistical analysis

All statistical analyses were performed in Matlab R2024b (MathWorks, Natick, MA, USA) using two-tailed tests. Normality was visually assessed using histograms and Q–Q plots. Formal tests like the Shapiro–Wilk test was not used to avoid false positives in a moderately sized sample.

Previous research in this cohort found that age was negatively correlated with striatal K_i_^cer^ [53] and positively correlated with subcortical χ [32]. Given these relationships, and considering that NM-MRI signal increases with age [54] and that older FTD individuals show more pronounced frontotemporal and subcortical atrophy [55], age was included as a covariate in all models. Partial Pearson’s correlations were used when variables were approximately normally distributed. For analyses involving current medication, a categorical variable, one-way ANCOVA was performed when assumptions of normality and homogeneity of variance (Levene’s test) were met. We used a one-way ANOVA to assess the effect of ethnicity on each neurochemical measure in the patient sample. Ethnicity was treated as a categorical variable based on self-reported ethnicity and coded into 6 categories (White, East Asian, Black, Other, South Asian, White and Black).

For the primary analyses, bvFTD scores were correlated with striatal K_i_^cer^, SN-VTA NM-CNR, and χ in the striatum and SN-VTA. In additional exploratory analyses, bvFTD scores were correlated with K_i_^cer^ and χ in each of the striatal subregions, negative symptoms, and age of onset. Multiple comparisons for the exploratory tests were controlled using a false discovery rate (FDR) correction within each modality (clinical or neuroimaging technique).

As QSM and NM-MRI data were available in healthy controls, we conducted secondary case–control comparisons for ROIs in which a significant association was observed between bvFTD-like scores and either χ or NM-CNR in patients. Specifically, patients were stratified by bvFTD-like score, and χ or NM-CNR values in the highest quartile were compared with those of healthy controls for the corresponding ROI using independent-samples t-tests. To explore if striatal iron and SN-VTA iron are coupled in controls but dysregulated in patients with schizophrenia, we examined Pearson correlations between SN-VTA χ and striatal χ within each group. Group differences in correlation coefficients were assessed using a Fisher’s r-to-z transformation.

To directly compare bvFTD-like and non–bvFTD-like patients with schizophrenia, we conducted linear regression models with subgroup (bvFTD-like [*n* = 8] vs non–bvFTD-like [*n* = 68]) as the independent variable and age as a covariate. Primary neuroimaging measures (striatal K_i_^cer^, SN–VTA NM-CNR, striatal χ, and SN–VTA χ) were examined, followed by exploratory analyses of K_i_^cer^ and χ within individual striatal subregions.

#### Machine-learning analysis

To explore whether a multimodal combination of neuroimaging features could jointly predict the bvFTD-like structural pattern, an exploratory machine-learning analysis was performed in NeuroMiner v1.2 [52]. A support vector machine classifier, implemented within NeuroMiner’s LIBSVM library, was trained to distinguish patients with high versus low bvFTD scores (median split) using a nested cross-validation framework with 3 inner and outer folds and 10 permutations. Two sets of features were examined: (1) primary features set (*n* = 4): striatal K_i_^cer^, SN-VTA NM-CNR, SN-VTA χ and striatal χ; (2) subregion features set (*n* = 8): associative, limbic and sensorimotor striatal K_i_^cer^, caudate, putamen, nucleus accumbens, and substantia nigra χ and SN-VTA NM-CNR.

The analysis included all patients who had at least one of these features available (*n* = 76). Preprocessing took place within each fold to prevent data leakage: all features were scaled between –1 and 1, missing data were imputed using sequential k-nearest neighbors (k = 7), and scaling was then repeated.

The model was initially trained without covariates, and the decision scores were subsequently checked for associations with demographic or clinical variables (sex, age, age of onset, illness duration). Decision scores were significantly associated with age and sex. Therefore, models were re-run with age and sex included as nuisance covariates to account for their potential confounding effects.

In addition, to preserve the continuous variability of the bvFTD score, support vector regression (SVR; ε-SVR implemented in LIBSVM) was performed using the same feature sets, preprocessing pipeline, and nested cross-validation framework. Age and sex were included as covariates within cross-validation folds.

## Results

### Participants

A total of 159 participants (81 healthy controls and 78 with schizophrenia) underwent sMRI. Of these, two scans per group were excluded due to low image quality, leaving 76 patients and 79 controls in the analyzed dataset. Of the patients, 40 had usable [¹⁸F]-DOPA PET data (one excluded for excessive movement), 69 had QSM data (four excluded for movement and three for streaking artefacts), and 68 had NM-MRI data (three excluded for movement). Demographic, clinical, and imaging characteristics are summarized in Table 1. The mean age for patients was 31.2 (7.0), 71% of the sample were male, 32% were White, 40% Black, 12% Asian and 4% Mixed. Patients and controls did not differ significantly in age (*t* = 1.14, *p* = 0.26), sex (χ² = 0.29, *p* = 0.59), or ethnicity (χ² = 6.72, *p* = 0.57). Our one-way ANOVA identified no significant relationship between ethnicity and any neurochemical measure in the patient sample (all *p* > 0.05; Supplementary Table S1). The independent-samples *t*-test identified significantly higher bvFTD scores (mean = −0.505, SD = 0.438) compared to healthy controls (mean = –0.706, SD = 0.394; *t* = 2.996, *p* = 0.003, Cohen’s d = 0.48). This quantification indicates that, on average, schizophrenia patients show a modest shift toward the bvFTD structural pattern relative to controls.

### Application of the bvFTD classifier

The published bvFTD classifier [21] showed excellent discrimination when retrained in the FTD discovery cohort (area under the receiver operating characteristic curve [AUROC] = 0.94; balanced accuracy [BAC] = 88.1%; sensitivity = 84.1%; specificity = 92%). When applied to the combined schizophrenia and local healthy control dataset, the model yielded an AUROC of 0.64 and a BAC of 52.7% for distinguishing schizophrenia patients from healthy controls based on the bvFTD-derived decision function. Specificity was high (94.9%), whereas sensitivity was low (10.5%), indicating that only a small proportion of schizophrenia patients were classified as bvFTD-like. In total, eight of 76 patients with schizophrenia (10.5%) and four of 79 healthy controls (5.1%) exceeded the bvFTD threshold (see Supplementary Table S2 and Supplementary Fig. S1 for model performance details).

### Associations between bvFTD score and clinical measures

Age was included as a covariate in all analyses. The results from these are displayed in Table 2. bvFTD scores were not associated with PANSS negative symptoms (*r* = 0.121, pFDR = 0.511) or age of onset (*r* = –0.104, pFDR = 0.511), and did not differ significantly across medication groups (ANCOVA: *F* (7,67) = 1.547, pFDR = 0.511). Likewise, bvFTD scores did not differ between medicated and unmedicated patients (*t* = −1.043, *p* = 0.3). Finally, bvFTD scores were not associated with chlorpromazine daily equivalent doses (*r* = –0.042, pFDR = 0.719).

**Table 2:** Associations between bvFTD score and clinical and neurochemical measures.

Variables	Stat	*p*	*p*FDR
Clinical measures (*n* = 76)			
PANSS negative	*r* = 0.121	0.337	0.511
Age of onset, years	*r* = –0.104	0.383	0.511
Current medication	*F* (7,67) = 1.547	0.167	0.511
Chlorpromazine daily equivalent doses	*r* = −0.042	0.719	0.719
PET (*n* = 40)			
Striatum	*r* = –0.343	0.032*	Primary analysis
Associative	*r* = –0.377	0.018*	0.044*
Sensorimotor	*r* = –0.168	0.307	0.307
Limbic	*r* = –0.349	0.029*	0.044*
NM-MRI (*n* = 68)			
Substantia nigra	*r* = –0.202	0.102	Primary analysis
QSM (*n* = 69)			
Striatum	*r* = 0.282	0.020*	Primary analysis
Caudate nucleus	*r* = 0.159	0.196	0.294
Putamen	*r* = 0.301	0.013*	0.038*
Nucleus accumbens	*r* = 0.113	0.359	0.359
Substantia nigra	*r* = –0.015	0.903	Primary analysis

Partial Pearson correlation coefficients (r) were used for continuous variables, and one-way ANCOVA, both controlling for age, was used to compare bvFTD-likeness scores across medication groups. *p* values marked with * indicate statistical significance at *p* <0.05. False discovery rate (FDR) correction was applied separately within each modality (clinical, PET, QSM). NM-MRI included only a single variable and therefore was not subjected to FDR correction (“Primary analysis”).

### Associations between bvFTD score and striatal dopamine (K_i_^cer^)

**Fig. 1:**
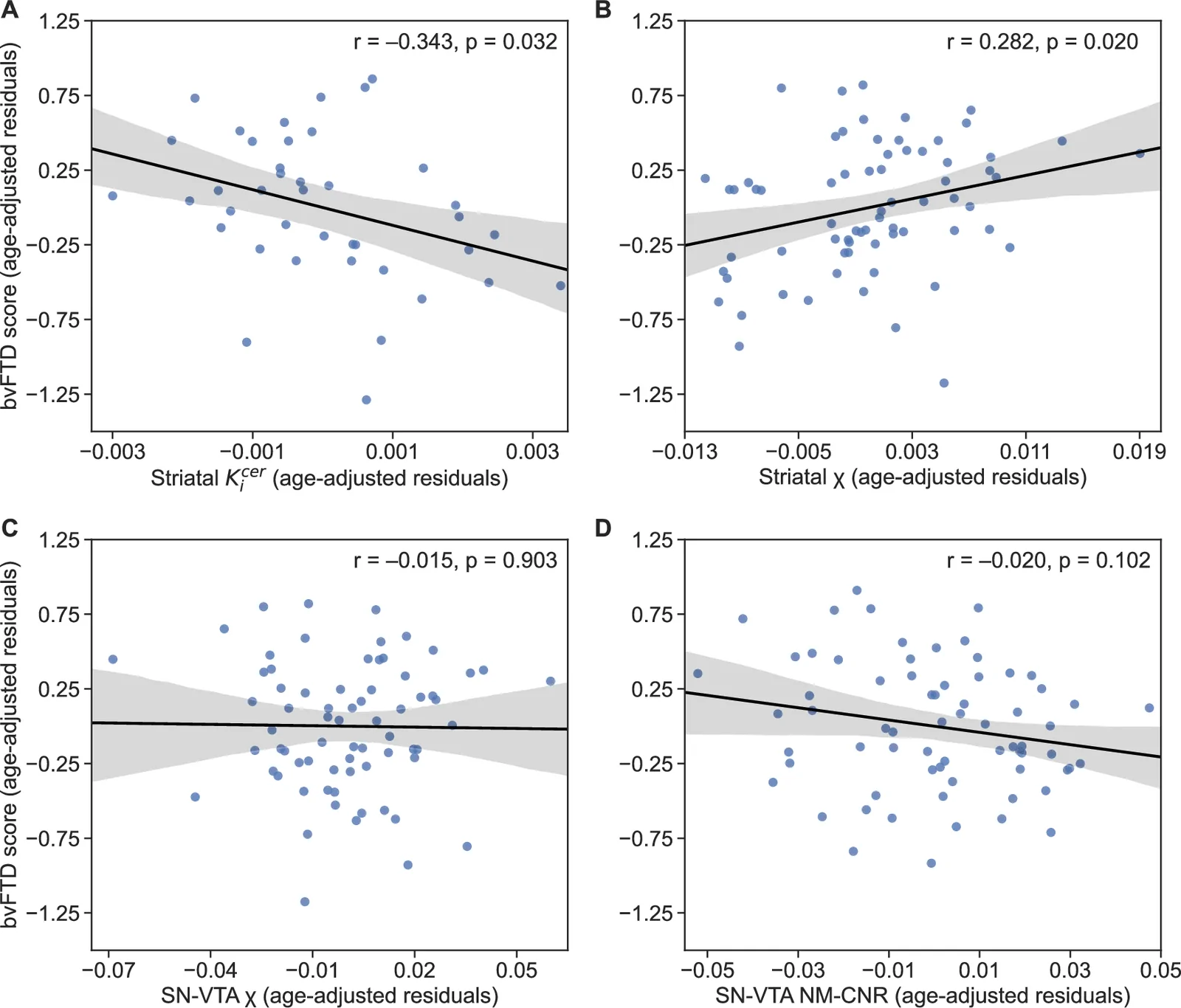
Correlations between bvFTD-like pattern expression and basal ganglia dopamine synthesis capacity (K_i_^cer^), magnetic susceptibility (χ), and neuromelanin-sensitive MRI contrast-to-noise ratio (NM-CNR). Scatter plots depicting relationships between bvFTD score and **A** striatal K_i_^cer^, **B** striatal χ, **C** substantia nigra and ventral tegmental area (SN-VTA) χ, (**D**) and SN-VTA NM-CNR. Each point represents one participant with schizophrenia.

Striatal K_i_^cer^ showed a significant negative correlation with bvFTD scores in our primary PET analysis (*r* = –0.343; *p* = 0.032; Fig. 1A). In the exploratory subregion analysis, bvFTD scores showed significant negative correlations with K_i_^cer^ in associative (*r* = –0.377; pFDR = 0.044) and limbic (*r* = –0.349; pFDR = 0.044) but not sensorimotor subregions (*r* = –0.168; pFDR = 0.307).

### Associations between bvFTD score and basal ganglia QSM

In the primary QSM analyses, bvFTD scores had a significant positive correlation with striatal χ (*r* = 0.282, *p* = 0.020; Fig. 1B) but no significant relationship with SN-VTA χ (*r* = –0.015, *p* = 0.903; Fig. 1C). In the exploratory striatal subregion analysis, bvFTD scores only showed a significant positive correlation with χ in the putamen (*r* = 0.301, pFDR = 0.013), but not in the caudate nucleus (*r* = 0.159, pFDR = 0.294) or nucleus accumbens (*r* = 0.113, pFDR = 0.359).

Additionally, we showed that striatal χ was non-significantly higher for patients in the highest quartile of bvFTD-like scores (mean = 33.956, SD = 6.391) compared to controls (mean = 31.827, SD = 7.576; *t* = 1.204, *p* = 0.239). While we did not identify a correlation between SN-VTA χ and striatal χ in the patients (*r* = 0.159 and *p* = 0.193) a positive correlation was identified in the controls (*r* = 0.321 and *p* = 0.004); however, the difference between these correlation coefficients did not reach statistical significance on direct comparison (*z* = 1.029 and *p* = 0.304).

### Associations between bvFTD score and SN-VTA NM-CNR

NM-CNR did not significantly correlate with bvFTD score (*r* = –0.202, *p* = 0.102; Fig. 1D).

### Group differences between bvFTD-like and non-bvFTD-like patients

No statistically significant group differences were observed (Supplementary Table S3). However, effect directions were broadly consistent with the continuous analyses, with bvFTD-like patients showing numerically lower striatal dopamine synthesis and higher putaminal χ relative to the remainder of the cohort.

### Exploratory machine-learning analysis

An exploratory machine-learning analysis was conducted to test whether multimodal neurochemical features could discriminate patients with high versus low bvFTD scores (median split). Across models, classification performance was at chance level (Supplementary Table S4).

Using the primary feature set—striatum K_i_^cer^, substantia nigra NM-MRI contrast, striatal χ, and substantia nigra χ—the classifier achieved AUROC = 0.62 and BAC = 58.7%. The subregion feature set (associative, limbic, sensorimotor K_i_^cer^; caudate, putamen, nucleus accumbens, SN-VTA χ, and SN-VTA NM-MRI) similarly performed at chance (AUROC = 0.59; BAC = 54.7%).

Decision scores showed an association with age and sex, and therefore models were re-run with age and sex included as nuisance covariates. However, covariate adjustment did not improve performance (e.g., primary-feature model: AUROC = 0.64; BAC = 62.7%).

In addition, SVR models also performed at chance level (Supplementary Table S5; primary-feature model: *r* = 0.22, 95% CI −0.01 to 0.43; subregion-feature model: *r* = 0.13, 95% CI −0.10 to 0.35), indicating that multimodal neurochemical features did not reliably predict variance in bvFTD-like structural expression.

## Discussion

This study examined whether a bvFTD-like structural brain pattern in schizophrenia is associated with distinct neurochemical or clinical features, to better characterize heterogeneity within schizophrenia. Importantly, our interpretation is framed at the level of large-scale brain networks rather than specific molecular or proteinopathic mechanisms. Higher bvFTD scores correlated inversely with striatal dopamine (K_i_^cer^) and positively with striatal iron (QSM χ), whereas no associations were detected with midbrain neuromelanin (SN-VTA NM-CNR), a byproduct of dopamine synthesis, midbrain iron (SN-VTA χ), or clinical measures. These findings extend previous work showing overlaps between schizophrenia and bvFTD [16, 21] and present the first evidence that a subset of schizophrenia patients with structural brain similarities to bvFTD may also have distinct neurochemical features.

The relatively low sensitivity (10.5%) of the classifier in schizophrenia should be interpreted cautiously. As the classifier was originally trained to distinguish bvFTD from healthy controls [21] rather than to detect a subtype within schizophrenia, reduced sensitivity likely reflects that only a minority of patients express a bvFTD-like pattern. Importantly, subsequent analyses relied on the continuous bvFTD score, allowing dimensional associations independent of binary classification.

### Dopaminergic findings: links to a hypodopaminergic pattern

The inverse association between the bvFTD-like pattern and striatal dopamine was strongest in the associative and limbic striatal subdivisions, which receive dense inputs from prefrontal and limbic cortices [56]. In bvFTD, lower striatal dopamine transporter binding has been reported [22, 57]. While direct studies remain limited, reduced striatal dopamine synthesis has also been associated with apathy [58], which is a hallmark symptom of bvFTD [59]. Moreover, behavioral features such as impulsivity and cognitive impairment have been linked to dysfunction in prefrontal and limbic cortices [60–62], which project to associative and limbic striatum [56]. Schizophrenia, by contrast, is typically characterized by elevated striatal dopamine synthesis [24], but increasing evidence points to a subgroup—sometimes termed “Type B”—with normal or reduced dopamine, prominent negative symptoms, and D2 receptor blocker antipsychotic treatment resistance [25, 26, 63]. These findings suggest that the bvFTD-like pattern may better align with the hypodopaminergic profile seen in bvFTD, potentially reflecting a distinct pathophysiological mechanism within schizophrenia. Since antipsychotic medications primarily target the dopaminergic system [63], it is possible that these individuals may require differential treatment.

### Network-level mechanisms: corticostriatal circuit involvement

One explanation for these differences is that schizophrenia patients with a bvFTD-like structural pattern might share network-level pathophysiological features with bvFTD, such as neurodegenerative changes affecting corticostriatal (or corticostriatothalamic) circuits. The frontal cortex regulates midbrain dopamine neurons through these pathways [64, 65], so alteration in key regions—such as the medial prefrontal cortex and anterior cingulate—may weaken excitatory input to dopamine-producing cells and reduce striatal dopamine synthesis. Degeneration within these circuits may involve loss of dopaminergic projections or reduced metabolic connectivity [23, 66, 67] as reported in FTD. However, no associations were observed between bvFTD-likeness and SN–VTA QSM or NM-MRI measures, suggesting that abnormalities in this subgroup are primarily expressed at the level of striatal terminals rather than midbrain cell bodies.

### Regional specificity within the striatum

The regional specificity of our PET findings—strongest associations in the associative and limbic striatum, and absent in the sensorimotor striatum—may reflect the anatomical topography of bvFTD-related pathology. bvFTD predominantly affects prefrontal and limbic cortices [21, 68], which preferentially project to associative and limbic striatal territories [56], whereas the sensorimotor striatum receives major inputs from motor cortices [56] that are relatively spared in bvFTD [69]. Accordingly, if the bvFTD-like structural phenotype reflects disruption of fronto–limbic–striatal circuits, dopaminergic alterations would be expected to manifest primarily in associative and limbic regions. Importantly, these findings do not contradict the established hyperdopaminergic model of schizophrenia but instead highlight circuit-specific heterogeneity within the disorder.

### Iron accumulation: a potential link to neurodegeneration

Beyond dopaminergic alterations, we observed a positive correlation between bvFTD-like scores and striatal iron, driven specifically by the putamen. In vivo and postmortem MRI studies have linked FTD to striatal iron accumulation [70–72]: these associations were strongest in the putamen [72], where iron levels correlated with behavioral disinhibition [72]. Protein aggregation or mitochondrial dysfunction are thought to drive iron accumulation in bvFTD, promoting neuronal death via oxidative damage and ferroptosis [11]. Postmortem evidence in schizophrenia similarly implicates protein accumulation and ubiquitin–proteasome system dysfunction [73], and a recent large case–control study reported increased iron and reduced ferritin in the prefrontal cortex [74]. Because ferritin sequesters iron and limits its redox activity [27], increased chemically active iron could promote bvFTD-like neurodegenerative processes in schizophrenia. Thus, the positive association between bvFTD-like score and striatal χ may reflect iron accumulation that contributes to both neurodegeneration and hypodopaminergia.

These findings add to the evidence highlighting the putamen as a region of special interest in QSM analyses in schizophrenia. Meta-analysis of QSM studies in psychosis has identified a large, significant heterogeneity in putaminal χ (I² = 87%; *p* < 0·001), driven by lower χ in first-episode psychosis but higher values in chronic schizophrenia [28]. This pattern could be explained by the presence of a bvFTD-like subgroup in which progressive striatal iron accumulation contributes to neurodegeneration and reduced dopamine synthesis. Notably, our earlier case–control analyses in this sample showed lower mean striatal χ in those with schizophrenia relative to controls [32]. In this context, the present finding of a positive association between bvFTD-likeness and higher striatal χ within patients may reflect heterogeneity in iron-related alterations, potentially attributable to neurodevelopmental (low iron) and neurodegenerative (high iron) disease subtypes. Although patients in the highest quartile of bvFTD-like scores exhibited numerically higher striatal χ than controls, this difference did not reach statistical significance, possibly due to low sample size in that subgroup. A case–control study comparing striatal χ in bvFTD patients with matched healthy controls will be required to directly test the hypothesis that this neurodegenerative condition is associated with striatal iron accumulation. It should also be noted that the patients in our cohort had a much shorter mean duration of illness (2.7 years - see Table 1) than participants in the original paper [21]. The bvFTD signature and potential iron accumulation may therefore be less pronounced at early stages of schizophrenia compared to more chronic presentations.

### Iron coupling between midbrain and striatum

The presence of a significant positive correlation between striatal χ and SN–VTA χ in healthy controls is consistent with coordinated regulation of iron across the basal ganglia. The lack of such a correlation in patients with schizophrenia suggests that basal ganglia iron dysregulation may exist in the condition. However, the difference between these correlations was not significantly different, indicating that a larger sample may be required to fully examine this hypothesis.

### Clinical measures: lack of association with negative symptoms

The lack of a correlation between bvFTD-like patterns and negative symptom severity was unexpected, as frontotemporal alterations are often linked to apathy and social withdrawal [75, 76]. One explanation may relate to our sample characteristics: all patients were receiving standard antipsychotics, and none required clozapine, suggesting that most were treatment responsive. Negative symptoms tend to be more prominent in chronic, treatment-resistant illness [77], where bvFTD-like alterations may have greater clinical relevance. This finding also contrasts with previous work reporting such an association [21]. Notably, that cohort had a longer illness duration (mean ≥4.5 years) than our sample (mean = 2.7 years), suggesting that stage-related differences may explain the discrepancy. The PANSS negative subscale may also fail to capture overlapping features such as apathy or disinhibition.

### Genetic and pathological considerations

bvFTD is a clinico-imaging syndrome associated with heterogeneous underlying pathologies, including FTLD-tau and FTLD-TDP [12]. The bvFTD classifier applied in this study was developed in clinically diagnosed bvFTD cohorts without neuropathological stratification, and the existing literature examining structural overlap between schizophrenia and bvFTD has likewise focused on syndrome-level imaging patterns rather than pathology-defined FTLD subtypes [16, 21]. Nevertheless, shared genetic variants from previous studies between bvFTD and schizophrenia might suggest potential mechanisms. The C9orf29 gene is shared between schizophrenia and bvFTD [78]: in bvFTD this gene is associated with more psychotic symptoms and more “schizophrenia-like” brain phenotype [21]. The C9orf72 protein is a moderator of autophagy and inflammation [79] and is known to activate proinflammatory pathways along the gut-brain axis thus potentially causing neuroinflammation that leads to iron accumulation and neurodegeneration through microglial disruptions [18, 80]. MAPT is another shared risk gene: this gene produces Microtubule-Associated Protein Tau, which is crucial in the production of tau protein [81]. Tau aggregation occurs in several neurodegenerative disorders including Alzheimer’s disease and FTD [81], and is associated with striatal dopaminergic dysfunction and iron accumulation in the latter [82–84]. However, a recent meta-analysis finds tau is generally decreased in schizophrenia [85]: this may be due to only a small number of schizophrenia patients experiencing tauopathy or potential non-tau related mechanisms of action [86].

### Limitations

Several limitations should be considered. Sample size was modest, especially in the multimodal subsamples, which may have obscured small effects. The cross-sectional design precludes causal inference regarding temporal relationships between structural and neurochemical alterations and does not allow conclusions about possible progression from schizophrenia to bvFTD. We did not assess longitudinal outcomes or neuropathology, and therefore cannot determine whether the observed phenotype reflects stable heterogeneity or potential neurodegenerative risk. The exploratory machine-learning analysis combining multimodal neurochemical variables performed at chance level, indicating that in this moderate sample, dopaminergic and iron-related measures did not jointly account for variance in bvFTD-like structural expression. There was no data on treatment response, so associations between bvFTD-like alterations and treatment resistance could not be examined. We did not have striatal dopamine measures in healthy controls, so we were not able to run a comparison with bvFTD scores to see if it was specific to schizophrenia. Finally, we did not collect genetic data or systematic family history information, including family history of FTD—a factor of potential relevance given the increased risk of schizophrenia in first-degree relatives of FTD patients [87]. Future studies incorporating such data are needed to determine whether the bvFTD-like phenotype is associated with shared genetic risk factors (e.g., C9orf72) or family history of neurodegenerative disease.

### Future directions

Future research could focus on treatment-resistant schizophrenia, where bvFTD-like brain alterations may be more prevalent and dopaminergic function diverges from the typical pattern [25, 26]. Longitudinal studies following high-risk or first-episode cohorts, incorporating biomarker-based characterization and detailed assessments of apathy, disinhibition, and cognitive function, could clarify whether bvFTD-like brain alterations in schizophrenia are associated with longitudinal changes in clinical course or neurobiological markers potentially relevant to neurodegenerative processes. In addition, examining whether a subgroup of schizophrenia patients shows stronger overlap with specific FTLD subtypes may help elucidate mechanisms at the protein level.

## Conclusion

In conclusion, we provide preliminary evidence that a subset of individuals with schizophrenia express a bvFTD-like structural pattern that is associated with reduced dopamine synthesis and increased striatal iron. These findings suggest the existence of a dimensional phenotype characterized by neurodegeneration-like alterations within corticostriatal circuits. This profile differs from both the canonical hyperdopaminergic model and prior reports linking schizophrenia to low brain iron, highlighting the biological heterogeneity within the disorder. Future work will be required to determine whether this bvFTD-like dimension has implications for treatment response or clinical stratification.

## Supplementary information

REVISED Supplement

## Data Availability

Data were taken from a previous clinical trial (NCT04038957) as part of the Department of Psychosis Shared Database Initiative at the Institute for Psychiatry, Psychology and Neuroscience. For details on the data please reach out to Dr Luke Vano (luke.vano@kcl.ac.uk).
