# Supplementary material for: A frontotemporal dementia–like phenotype in schizophrenia: links to striatal dopamine and iron accumulation
Source: Neuropsychopharmacology. 2026 Apr 25;51(11):2074–82. doi: 10.1038/s41386-026-02426-x (PMC13598093; doi:10.1038/s41386-026-02426-x)
Supplement: Supplementary file 1 [file 41386_2026_2426_MOESM1_ESM.docx]

**Supplementary Materials**

**Table S1. Associations between ethnicity and neurochemical measures**

| **Variables** | **F stat** | **p** |
| --- | --- | --- |
| PET (n=40) |  |  |
| Striatum | 0.735 | 0.487 |
| Associative | 0.248 | 0.782 |
| Sensorimotor | 2.351 | 0.109 |
| Limbic | 0.059 | 0.943 |
| NM-MRI (n=68) |  |  |
| Substantia nigra | 0.510 | 0.603 |
| QSM (n=69) |  |  |
| Striatum | 1.494 | 0.232 |
| Caudate nucleus | 1.215 | 0.303 |
| Putamen | 1.168 | 0.317 |
| Nucleus accumbens | 1.422 | 0.249 |
| Substantia nigra | 0.980 | 0.381 |

Ethnicity was treated as a categorical variable (six self-reported categories) and assessed using one-way ANOVA.

**Table S2. Performance metrics of the bvFTD classifier in discovery and local cohorts**

|  | **AUROC (95%-CI)** | **BAC (%)** | **Sensitivity (%)** | | **Specificity (%)** | |
| --- | --- | --- | --- | --- | --- | --- |
| Discovery Cohort | 0.94 | 88.1 | 84.1 | 92 | |  |
| Local Schizophrenia Cohort | 0.64 | 52.7 | 10.5 | 94.9 | |  |

Model performance in discovery cohort was reproduced within the original bvFTD discovery sample (15). All performance metrics for the local cohort were computed using the same linear SVM model externally applied to our schizophrenia sample.

**Figure S1.**

**Figure S1. ROC Curves for bvFTD classifier performance in discovery and local cohorts**. Receiver Operating Characteristic (ROC) curves showing the performance of the bvFTD classifier in the discovery cohort (blue) and external application to the combined schizophrenia and local healthy control sample (red). The classifier demonstrated excellent discrimination between bvFTD patients and healthy controls in the discovery sample (AUROC = 0.94), but poor discrimination when distinguishing schizophrenia patients from local healthy controls (AUROC = 0.64). The dashed diagonal line indicates chance-level classification (AUROC = 0.50).

**Table S3. Group differences between bvFTD-like and non–bvFTD-like patients (adjusted for age)**

| **Variables** | **β (SE), ×10⁻³** | **t** | ***p*** |
| --- | --- | --- | --- |
| PET (n=40) |  |  |  |
| Striatum | −0.423 (0.596) | −0.71 | 0.483 |
| Associative | −0.806 (0.689) | −1.17 | 0.250 |
| Sensorimotor | 0.300 (0.731) | 0.41 | 0.684 |
| Limbic | −1.291 (0.735) | −1.76 | 0.088 |
| NM-MRI (n=68) |  |  |  |
| Substantia nigra | −13.686 (8.276) | −1.65 | 0.103 |
| QSM (n=69) |  |  |  |
| Striatum | 0.119 (2.715) | 0.04 | 0.965 |
| Caudate nucleus | −4.603 (3.187) | −1.44 | 0.153 |
| Putamen | 3.335 (3.176) | 1.05 | 0.297 |
| Nucleus accumbens | 4.447 (5.479) | 0.81 | 0.420 |
| Substantia nigra | −10.650 (9.175) | −1.16 | 0.250 |

Group differences in each neurochemical measure were assessed using linear regression models with age included as a covariate.

**Table S4. Performance metrics for exploratory machine-learning classification models**

| **Model** | **Covariates** | **Features** | **AUROC** | **BAC (%)** | **Sensitivity (%)** | **Specificity (%)** |
| --- | --- | --- | --- | --- | --- | --- |
| Model 1 | - | 4 primary features | 0.62 | 58.7 | 59.5 | 57.9 |
| Model 2 | - | 8 subregion features | 0.59 | 54.7 | 59.5 | 50.0 |
| Model 3 | Age,Sex | 4 primary features | 0.64 | 62.7 | 62.2 | 63.2 |
| Model 4 | Age,Sex | 8 subregion features | 0.54 | 51.9 | 43.2 | 60.5 |

Performance metrics from exploratory SVM analyses distinguishing high vs. low bvFTD score groups (median split). All models performed at chance level.

**Table S5. Performance metrics for exploratory machine-learning regression models**

| **Model** | **Covariates** | **Features** | **MAE** | **MSE** | **r (95%-CI)** |
| --- | --- | --- | --- | --- | --- |
| Model 1 | Age,Sex | 4 primary features | 0.3 | 0.2 | 0.22 (-0.01-0.43) |
| Model 2 | Age,Sex | 8 subregion features | 0.4 | 0.2 | 0.13 (-0.10-0.35) |
|  |  |  |  |  |  |

Performance metrics from exploratory SVR analyses predicting bvFTD score. All models performed at chance level.

**Supplementary analysis: standardized effect sizes for structural differences.**

To quantify the magnitude of cortical atrophy in schizophrenia, we calculated standardized voxelwise effect sizes (Cohen’s d) for the schizophrenia versus healthy control grey matter volume (GMV) comparison using the same structural MRI data used in the classifier analysis. Structural images were preprocessed using the Computational Anatomy Toolbox (CAT12) implemented in SPM12, producing modulated and normalized GMV maps for each participant.

Group differences in GMV were assessed using a voxelwise two-sample t-test in SPM12 comparing schizophrenia patients and healthy controls . The model was specified using the standard VBM workflow with independent groups and unequal variance assumptions, statistical maps were thresholded at p < 0.001.

To provide standardized estimates of the magnitude of GMV differences, voxelwise effect sizes were derived from the resulting t-statistics using the standard conversion:

d = t × √(1/n_SZ + 1/n_HC).

where t represents the voxelwise t-statistic from the schizophrenia versus control comparison and n_{SZ} and n_{HC} correspond to the number of schizophrenia patients and healthy controls, respectively. The resulting Cohen’s d map therefore reflects the standardized magnitude of GMV differences across the brain.

Using this approach, the peak voxelwise effect size observed in frontoinsular regions was approximately d ≈ 0.8, indicating moderate-to-large GMV reductions in schizophrenia relative to controls.
